# Smiles as a Signal of Prosocial Behaviors Toward the Robot in the Therapeutic Setting for Children With Autism Spectrum Disorder

**DOI:** 10.3389/frobt.2021.599755

**Published:** 2021-05-26

**Authors:** SunKyoung Kim, Masakazu Hirokawa, Soichiro Matsuda, Atsushi Funahashi, Kenji Suzuki

**Affiliations:** ^1^Faculty of Engineering, Information and Systems, University of Tsukuba, Tsukuba, Japan; ^2^Faculty of Human Sciences, University of Tsukuba, Tsukuba, Japan; ^3^Faculty of Sport Science, Nippon Sport Science University, Yokohama, Japan

**Keywords:** smile, prosocial behavior, robot-assisted therapy, NAO, Bayesian model, electromyogram

## Abstract

We explored how robot-assisted therapy based on smile analysis may facilitate the prosocial behaviors of children with autism spectrum disorder. Prosocial behaviors, which are actions for the benefit of others, are required to belong to society and increase the quality of life. As smiling is a candidate for predicting prosocial behaviors in robot-assisted therapy, we measured smiles by annotating behaviors that were recorded with video cameras and by classifying facial muscle activities recorded with a wearable device. While interacting with a robot, the participants experienced two situations where participants' prosocial behaviors are expected, which were supporting the robot to walk and helping the robot from falling. We first explored the overall smiles at specific timings and prosocial behaviors. Then, we explored the smiles triggered by a robot and behavior changes before engaging in prosocial behaviors. The results show that the specific timing of smiles and prosocial behaviors increased in the second session of children with autism spectrum disorder. Additionally, a smile was followed by a series of behaviors before prosocial behavior. With a proposed Bayesian model, smiling, or heading predicted prosocial behaviors with higher accuracy compared to other variables. Particularly, voluntary prosocial behaviors were observed after smiling. The findings of this exploratory study imply that smiles might be a signal of prosocial behaviors. We also suggest a probabilistic model for predicting prosocial behaviors based on smile analysis, which could be applied to personalized robot-assisted therapy by controlling a robot's movements to arouse smiles and increase the probability that a child with autism spectrum disorder will engage in prosocial behaviors.

## 1. Introduction

Robotics have advanced and interactive robots have begun to be made available for a variety of purposes. Accordingly, some researchers have explored the benefits of using robots in therapeutic settings for children with autism spectrum disorder (ASD) (Cabibihan et al., 2013; Huijnen et al., 2018). As the main characteristic of ASD includes a lack of social skills (APA, 2013), robots have been applied in social contexts to facilitate fundamental behaviors for communicating and interacting with others (Pennisi et al., 2016). Researchers have reported that children with ASD show improved behaviors, such as increased eye contact and imitation while interacting with robots (Zheng et al., 2016; Cao et al., 2019). However, the ways that interactions with robots increase specific behaviors of children with ASD has not been fully investigated, and past studies have been limited to targeting basic social skills and behaviors. Therefore, to examine some ways robots may have further therapeutic potential for children with ASD, we designed this novel exploratory study. Smiling was used as a measurable signal of behavior change in therapeutic settings for children with ASD to investigate how robot-assisted therapy may facilitate prosocial behaviors based on smile analysis.

There are possible advantages to including the analysis of smiles in robot-assisted therapy for children with ASD. First, smiling is an innate nonverbal behavior (Shrout and Fiske, 1981; Rashotte, 2002; Parlade et al., 2009). An infant's first involuntarily smiles using mouth corners can be seen during the neonatal period. In the fourth week following birth, they can smile actively by moving muscles around their lips and eyes (Sroufe and Waters, 1976; Messinger et al., 1999). The contractions of specific facial muscles—the orbicularis oculi and zygomaticus major—have been observed when infants, as well as adults, are in a good mood. This muscle activity is accompanied by changes around the lips and eyes (Frank et al., 1993; Parlade et al., 2009). Although children with ASD have difficulty recognizing the smiles of others, they can exhibit voluntary smiles using those muscles (Hermelin and O'Connor, 1985; Sato, 2017).

Moreover, smiles can provide social and emotional information (Rashotte, 2002; Martin et al., 2017). The meanings of smiles differ depending on social situations, and the interpretation of other behaviors before, during, or after smiles can vary (Messinger et al., 2001). For instance, smiling when talking about positive things can be explained differently than smiling when talking about negative things (Sonnby-Borgström, 2002). This characteristic of smiles provides additional information for understanding other behaviors. Also, smiles may provide a criterion for evaluating the current developmental stage and progress in children with ASD (Funahashi et al., 2014; Samad et al., 2018).

Lastly, smiles may be a predictor of positive behaviors. Prosocial behaviors are actions that can benefit others, such as helping (Warneken and Tomasello, 2009), cooperating (Brownell, 2013), sharing resources (Dunfield, 2014), or providing emotional support (Svetlova et al., 2010). In previous studies, prosocial behaviors have been investigated in combination with positive moods (Carlson et al., 1988; Guéguen and De Gail, 2003; Telle and Pfister, 2016), and smiles were considered as an indicator of positive mood (Cunnigham, 1979; Baron, 1997; Forgas, 2002; Drouvelis and Grosskopf, 2016). Participants in the studies were willing to pick up a dropped pen, give change for a dollar, and play a game cooperatively after smiling. These findings suggest that people tend to engage in prosocial behaviors after they smile.

Learning prosocial behaviors is important for all children. Considering the personal advantages of receiving help from others and the social benefits of engaging in prosocial behaviors toward others, it is necessary for children with ASD to develop prosocial behaviors. Although the developmental sequence and timing have varied in previous studies, it has been reported that children with ASD can demonstrate prosocial behaviors. Action-based prosocial behaviors, such as picking up and returning items someone has dropped, have been observed in children with ASD between 24 and 60 months of age (Liebal et al., 2008). Also, emotion-based prosocial behaviors, such as responding to others' negative emotions, were reported in a study of 6- and 7-year-old children with ASD (Deschamps et al., 2014). As each child with ASD is in a different social developmental stage (APA, 2013), children with ASD need to practice various prosocial behaviors individually.

Robots could provide personalized therapy for children with ASD. Improvised interactions using a teleoperation method were applied to robot-assisted therapy (Thill et al., 2012; Hirokawa et al., 2018). In those studies, a robot's movements were controlled depending on a child's responses. In this research, we teleoperated a small humanoid robot called NAO (SoftBank Robotics Corp., Paris, France) and observed child–robot interactions in a therapeutic setting. The NAO robot is known for its use in education and therapy (Diehl et al., 2012; Ismail et al., 2012; Bharatharaj et al., 2017; Huijnen et al., 2017). In particular, it can play various roles as either a trainer or a peer of children with ASD. Additionally, using a NAO in the role of care-receiver in the classroom has been suggested for 3- to 6-year-old children to help them learn new words (Tanaka and Matsuzoe, 2012). Therefore, we assigned the care-receiving role to a NAO robot to create a social context in which children with ASD can practice prosocial behaviors. We sought to examine the children's behaviors with the robot when it walked around or fell down. We assumed that each child might smile before engaging in prosocial behaviors.

Thus, the purpose of this study was to explore the potential of personalized robot-assisted therapy based on smile detection for facilitating prosocial behaviors in therapy. The research was guided by the following research question:

Q. Are smiles a potential key factor in predicting prosocial behaviors in walking and falling situations with a robot?

To explore the research question, we adopted video analysis and a physiological signal-based method in a therapeutic setting; participants included children with ASD and typically developing (TD) children. The data obtained regarding TD children were used when observing the behavioral patterns of children with ASD to determine if they are the same. We first measured the duration of smiles and prosocial behaviors through video observation. Second, we complemented data regarding unobserved smiles with electromyogram (EMG) data from each participant. Third, we observed changes in smiles and prosocial behaviors. Finally, we applied a Bayesian framework with conditional probability to explore the potential of smiling as a predictive factor of prosocial behaviors as follows: If the occurrence of prosocial behaviors changes when smiles appear and the Bayesian model shows that smiles have predictability potential, this exploratory study may suggest a new framework for personalized robot-assisted therapy.

## 2. Materials and Methods

### 2.1. Participants

For this exploratory study, we recruited six children identified as having mild to moderate levels of ASD through the Institute for Developmental Research at the Aichi Human Service Center in Japan. For comparison, six TD children were also recruited. Children with ASD participated in four sessions, and TD children participated in three sessions of robot-assisted activities directed by a therapist. However, we were not able to include all the sessions due to the limitations involved with making the robot fall. Hence, we employed two sessions of children with ASD and one session of TD children; all of the sessions included both the robot walking situation and the robot falling situation. The average age of six children with ASD (four boys and two girls) was 9.67 years old (6–16, *SD* = 3.50) and the average age of six TD children (three boys and three girls) was 9.83 years old (6–11, *SD* = 2.04). None of the 12 children indicated they had any concerns about interacting with a robot and wearing a device. Table 1 shows the age information of the participants included in each session.

**Table 1:** Information on participants.

**Participant ID**	**Age**	**Analyzed session**
ASD-P1	16	Session 1, 2
ASD-P2	11	Session 1, 2
ASD-P3	8	Session 2, 4
ASD-P4	8	Session 2, 4
ASD-P5	6	Session 3, 4
ASD-P6	9	Session 1, 2
TD-P1	11	Session 2
TD-P2	11	Session 1
TD-P3	11	Session 3
TD-P4	9	Session 2
TD-P5	6	Session 1
TD-P6	11	Session 1

This research was approved by the Ethical Committee based on the Declaration of Helsinki and ethical rules established by the Aichi Human Service Center. The research data were collected in an intervention room of the same institute in compliance with the ethical principles. All caregivers of the children agreed to written informed consent and participated in the entire session.

### 2.2. Robot

A NAO robot was adopted to create social situations. It is a small-sized (58 cm in height) humanoid robot. NAO has been applied for therapy, rehabilitation, and education contexts requiring interactions with humans (Ismail et al., 2012; Tanaka and Matsuzoe, 2012; Pulido et al., 2017). It can communicate by expressing verbal and nonverbal behaviors. The 26 joints in the head, arms, legs, and pelvis of an NAO robot enable it to perform various motions, such as walking, sitting, and grasping. However, the movements are inflexible and unbalanced compared to human peers, which could lead children to perceive the robot as a care-receiver. After considering the functions and limitations of the NAO robot, we chose “walking with the robot” as the social context for this study. The expected social situations in the given scenario were (1) the robot walking, and (2) the robot falling; the desirable prosocial behaviors we looked for from the children were (1) helping the robot to walk, and (2) helping the robot stand up after it fell down. The NAO robot was controlled using teleoperated methods to create real-time interactions. In this study, we used the Wizard of OZ technique, a research method to make participants feel that they are interacting with an autonomous system (Riek, 2012). A human operator observed each child's responses to the NAO robot in the observation room and controlled the robot's movement by following the cues from a therapist in real time. The voice function of the robot was not used to make simplified interactions and to focus on nonverbal behaviors, which can affect prosocial behaviors.

### 2.3. Apparatus

**Figure 1:**
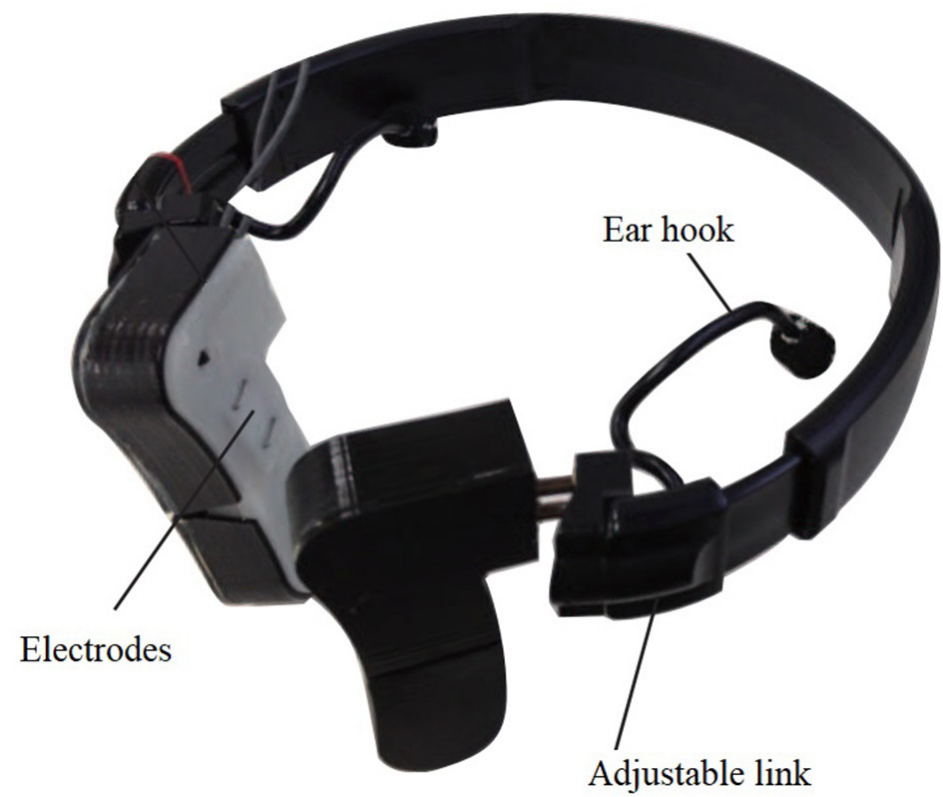
Smile Reader which was used in the robot-assisted therapy.

**Figure 2:**
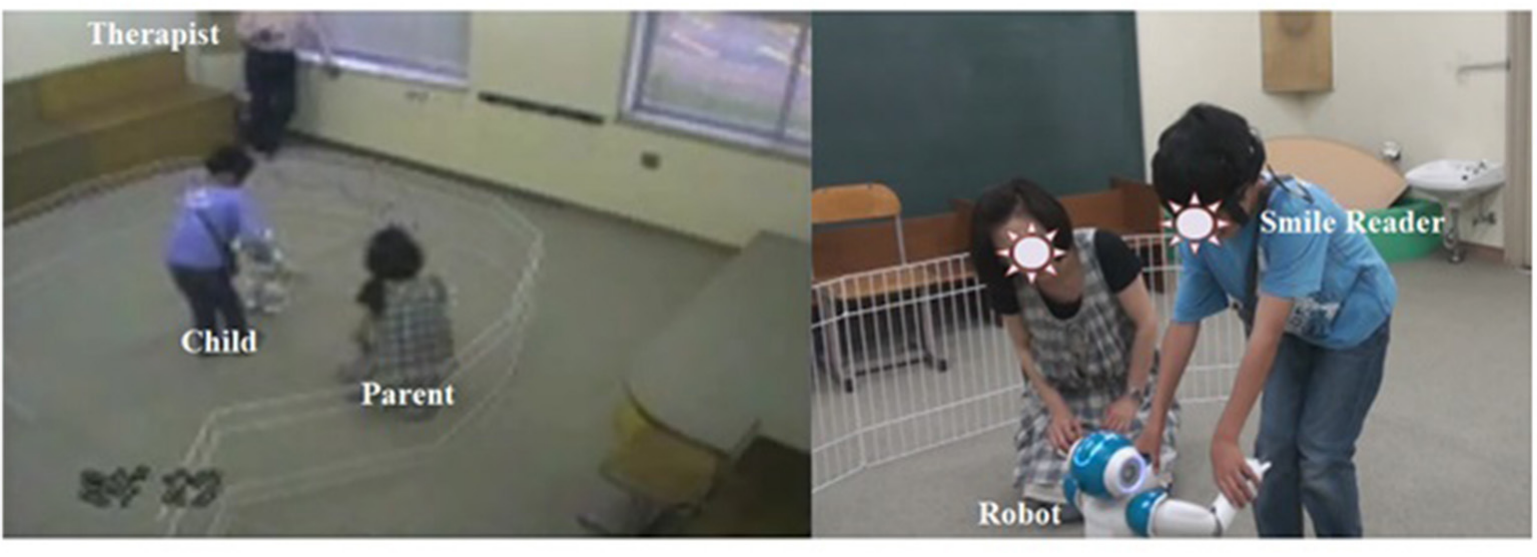
A child wearing a Smile Reader in the intervention room (captured by video cameras).

To analyze each participant's smiles and behaviors, video cameras and a wearable device, called the Smile Reader, were used in this research (Figures 1, 2). Four video cameras were installed on the ceiling of the intervention room. A therapist traced and captured each participant's movements with a hand-held video camera. The Smile Reader was used to record surface EMG from the facial muscles (Gruebler and Suzuki, 2014). The device was attached to both sides of the participant's face.

We used the wearable device with EMG sensors because it was designed and developed specifically for smile detection (Gruebler and Suzuki, 2014). This device can detect the contractions of facial muscles related to smiling—the orbicularis oculi and zygomaticus major. These facial muscle areas have been researched with EMG sensors to measure specific smiles that show spontaneous and positive emotions (Frank et al., 1993; Mauss and Robinson, 2009; Johnson et al., 2010; Perusquía-Hernández et al., 2019). Compared to other physiological sensors, such as electroencephalography and functional MRI, facial EMG can be attached directly to the facial muscles involved in smiling (Maria et al., 2019). Also, it can be used in both laboratory and therapy settings (Hirokawa et al., 2016). Furthermore, the performance evaluation of the Smile Reader has been investigated with adults in a laboratory and children with ASD in therapy; the device has proven reliability for accuracy in smile detection (Funahashi et al., 2014; Gruebler and Suzuki, 2014; Hirokawa et al., 2018).

In this research, each participant's facial EMG was recorded with the Smile Reader including four pairs of active electrodes and a BioLog (S&ME, Japan), a portable EMG logger that includes an amplifier. The devices were connected to a laptop wirelessly, and EMG signals were recorded in real time. To synchronize video and EMG data, a noticeable sign was included in the recorded EMG by using a time tagger.

### 2.4. Procedure

**Figure 3:**
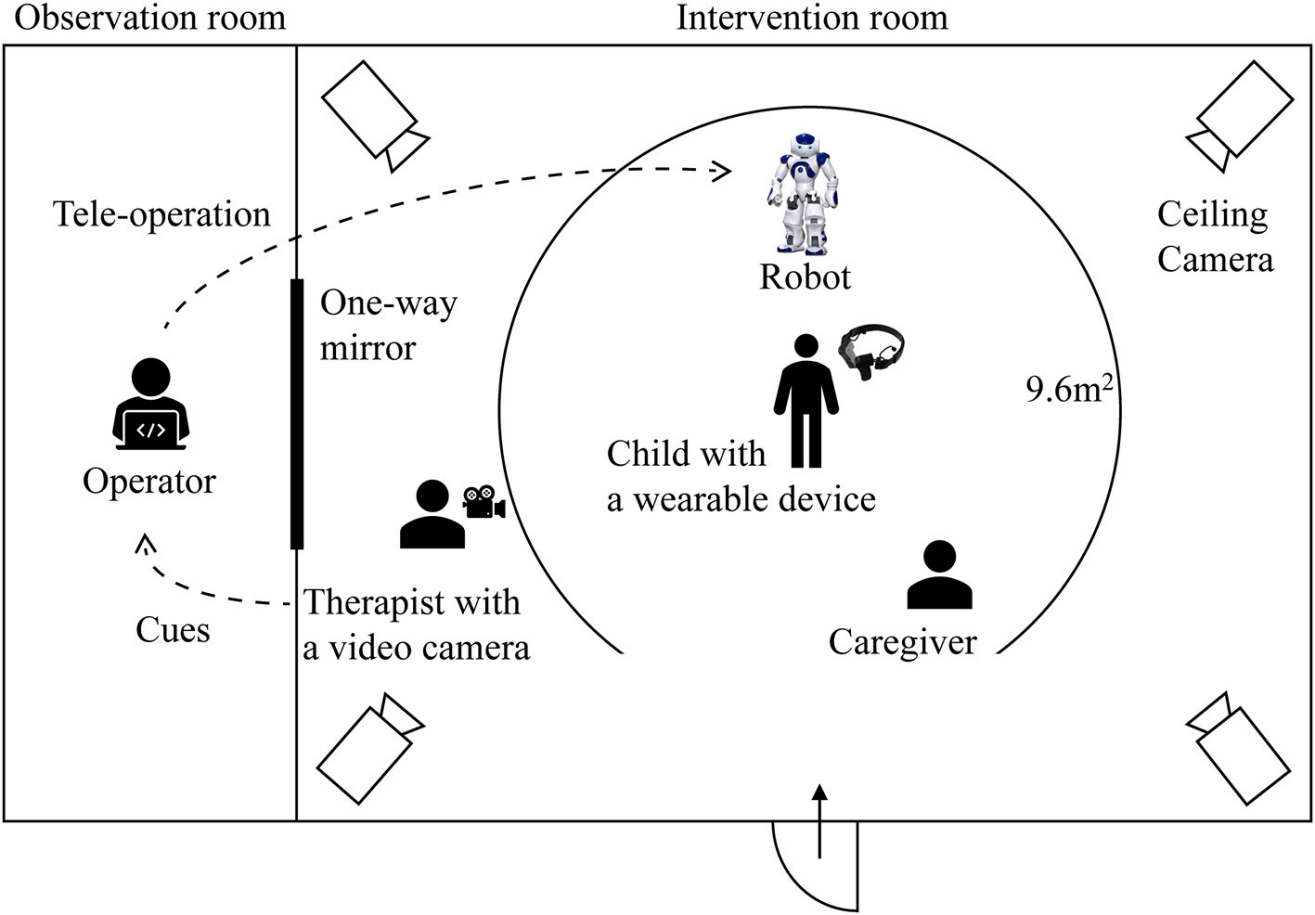
Overview of the experimental setting.

**Figure 4:**
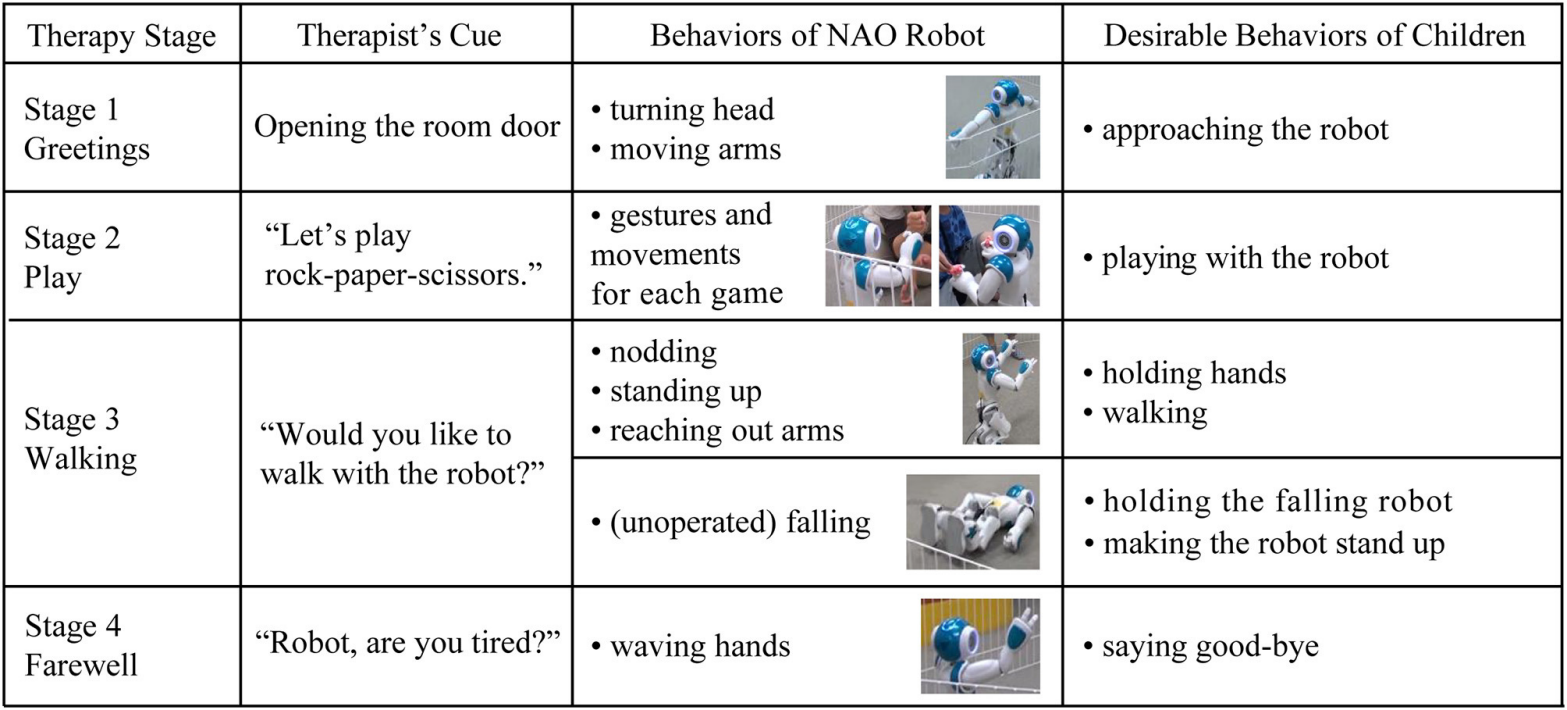
The designed behaviors of a NAO robot and desirable behaviors of children in each therapy stage.

This exploratory study is based on data collected during robot-assisted therapy. A NAO robot was used to assist a therapist in facilitating prosocial behavior of each child with ASD. The children with ASD participated in this research during the therapy. TD children who joined this research experienced the same procedure. Each child participated in a session every 2–3 weeks, a total of about 3 months. Each session lasted for 20–30 min, and every child was allowed to interact with the robot, a parent, or a therapist without restriction during all sessions. The 9.6 m^2^ area where each child could interact with the robot was fenced for safety (Figure 3). Their behaviors were recorded by ceiling cameras and the therapist's camera. Each therapy session was divided into four stages, and each stage included a specific cue from the therapist and the corresponding robot behaviors (Figure 4). When there were no cues from therapists, a human operator improvised the robot's movements.

The prescribed procedures of each stage are described in sections 2.4.1–2.4.5. The designed or anticipated behaviors and interactions related to the study were examined; improvised behaviors or interactions were excluded from video analysis.

#### 2.4.1. Preparation

Variables of this study were defined as follows: As in prior studies, smiles were defined as changes around the lips or eyes because the facial muscles related to positive affect are contracted by the changes (Frank et al., 1993; Parlade et al., 2009). Prosocial behaviors were defined differently in the two situations. In the walking situation, the children's prosocial behaviors included (1) approaching NAO to hold hand(s) and holding NAO's hand(s) or (2) walking together while holding NAO's hand(s). Prosocial behaviors during the falling of the NAO robot were defined as approaching NAO to hold its body and help the robot stand up.

Before interacting with a NAO robot, each participant was introduced to a preparation room and informed about the wearable device. While wearing the device that records facial muscle activities, each child was asked to watch 20 images that appeared on a computer screen. Each image appeared on the screen for 2 s. The images were emotionally neutral stimuli selected by a medical examiner, and they were used as a baseline to train the artificial neural network (ANN).

#### 2.4.2. Stage 1

Each child moved into an intervention room with a therapist and parent. The first stage began when the therapist pressed a button for a time tagger connected to EMG logging and opened the door of the intervention room. The therapist introduced each child to the robot, and the robot greeted them by moving its arms and turning its head to look around.

#### 2.4.3. Stage 2

In the second stage, each child interacted freely with the robot. In the middle of this stage, the therapist suggested the child and robot play a game of rock-paper-scissors or that they play catch by throwing and catching small beanbags. The NAO robot used hand gestures and body movements for each game. For example, during the rock-paper-scissors game, the robot made a handshape of rock, paper, or scissors, and when the robot won, it raised its arms. When the robot lost the game, it looked down and shook its head from side to side. When playing with the beanbags, the robot reached out its hands to receive the beanbags from a child and used its arms to throw them toward the child. Upon failing to catch a beanbag, the robot looked down, raised an arm, and tapped its own head.

#### 2.4.4. Stage 3

In the third stage, the therapist suggested walking together with the NAO robot, and the robot agreed by nodding, standing up, or reaching out with its arms. In this scenario, the desirable behaviors of children included holding the hands of the robot, and walking together. When a child did not show any expected behaviors, the therapist or a parent verbally directed the child to help the robot walk. However, when the robot fell by chance, the therapist observed each child's spontaneous responses without providing direction. The desirable expected behaviors of children were those that helped the robot stand up. When a child helped the robot to walk or stand up, the therapist said, “Thank you” to the child on behalf of the robot.

#### 2.4.5. Stage 4

In the last stage, the therapist suggested finishing the session. In response to the therapist's cue, the NAO nodded and waved a hand. After finishing the last stages, each child moved to the preparation room with a parent and took off the wearable device.

### 2.5. Video Analysis

Video analysis was adopted to measure the duration of smiles and prosocial behaviors.

#### 2.5.1. Step 1: Annotating Video Streams

To measure smiles and prosocial behaviors, each video was annotated based on the duration of each child's behaviors. The annotation included the beginning of the session, smiles, prosocial behaviors, other remarkable behaviors—such as waving hands, talking, and gesturing—and the unobservable facial expressions of each participant. The duration of smiles and prosocial behaviors were measured per millisecond (ms) by two trained examiners using Dartfish, a tagging software (Dartfish, Fribourg, Switzerland).

In the walking situation, the prosocial behaviors of children included (1) approaching NAO to hold hand(s) and holding NAO's hand(s) or (2) walking together while holding NAO's hand(s). We identified the point when a child started approaching NAO to hold hand(s) as the starting time of the prosocial behavior. Prosocial behaviors during the falling of the NAO robot were defined as approaching NAO to hold the body and helping the robot stand up. When the robot was falling in front of a child, holding the falling robot or making the robot stand up were defined as prosocial behaviors. We identified the point when a child released his or her hold on NAO's hand(s) or body as the ending time of prosocial behavior. The duration of prosocial behavior was calculated as the amount of time between the starting point and the ending point of the behavior. The duration of smiles was calculated as the amount of time between the starting time and ending time of the facial expression with upward lip corners or downward eye corners.

#### 2.5.2. Step 2: Selecting Segments of Video for Analysis

**Figure 5:**
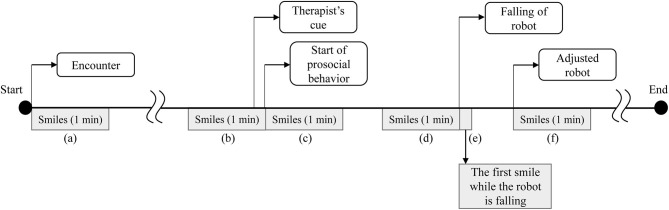
Timeline of analyzed parts of smiles.

To explore the specific timing of smiles and prosocial behaviors, six segments of the video were selected: (a) 1 min after entering the intervention room (encounter with the robot), (b) 1 min before starting prosocial behaviors in the walking situation, (c) 1 min after starting prosocial behaviors in the walking situation, (d) 1 min before starting prosocial behaviors in the falling situation, (e) the duration of the first smile when the robot is falling down, and (f) 1 min after the robot is adjusted in the falling situation (Figure 5). The segments were selected considering specific timings that might affect smiles and prosocial behaviors. Segments (b), (d), and (e) were selected considering that, in previous studies, prosocial behaviors occurred more frequently after smiling (Guéguen and De Gail, 2003; Vrugt and Vet, 2009). Segment (a) was selected considering that first impressions might change how a child behaved toward the robot throughout the session (Willis and Todorov, 2006). To explore if smiles before prosocial behaviors are more related to prosocial behaviors, (c) and (f)—smiles during or after prosocial behaviors—were selected. Each segment length was determined considering the duration of one type of activity, such as greeting the robot or playing rock-paper-scissors. Each activity lasted ~1 min. Also, the length was determined considering the duration of affect, including both emotion and mood (Beedie et al., 2005; Mauss and Robinson, 2009). As emotion is defined in seconds, shorter than mood, we considered a minimum duration of mood and a maximum duration of emotion.

Moreover, the analyzed timings were limited to the robot's first experience of walking and falling in a session, as each participant experienced a different number and duration of the social situations depending on participated sessions and interactions with the robot.

For the annotation of smiles in selected segments, reliability between the two examiners was high. The average intraclass correlation coefficient was 0.849 with 95% confidence interval from 0.811 to 0.879 [*F*_(307, 307)_ = 6.629, *p* < 0.001].

#### 2.5.3. Step 3: Observing Behavior Changes Before Engaging in Prosocial Behaviors

To explore how behavior changes happen after the robot's movement, 10 s of videos were selected before engaging in prosocial behaviors. The duration was selected by a study related to measuring affective engagement (Rudovic et al., 2017). There were four questions applied to the observations. First, are smiles observed before engaging in prosocial behaviors? Second, if a smile is observed, what triggered smiles? Third, what are the subsequent behaviors with smiles? Fourth, how are those behaviors linked to prosocial behaviors? To explore the questions, the head direction, facial expression, and body movement of each child were annotated every 1 s. The purpose of this observation was to investigate whether smiles can be triggered by a robot's movement and explore whether smiling is a potential predictive factor of prosocial behaviors.

#### 2.5.4. Step 4: Synchronizing EMG and Video Data

All the annotated video parts were synchronized with EMG data. As Stage 1 started by opening the intervention room door, and a therapist logged the moment into EMG data using a time tagger, we first checked the tag. Next, we synchronized the start timing of each annotation and the position of EMG. Lastly, we checked the synchronization in video streams.

### 2.6. EMG Signal Processing for Estimation of Unobserved Smiles

The smiles presented in this research are complemented by the durations of smiles detected by the EMG signal processing, as there were unobservable smiles. The ratio of unobservable parts in a whole session was a minimum of 2% and a maximum of 25% for a child with ASD, and a minimum of 3% and a maximum of 14% for a TD child. We used the EMG recordings from the wearable device to estimate smiles during the fragments unobservable with the video data. Based on this estimation, the duration of smiles was calculated. All results presented in this research were obtained from combined durations with the observable segments of video data and the unobservable segments with EMG data. We verified that none of the presented trends changed with the estimation of the EMG data.

To estimate unobserved smiles, cross-validation in machine learning was applied. We measured each child's facial EMG signals using four pairs of electrodes. When video cameras could not capture their face because children unexpectedly turned around or stood up, which were frequently included when doing prosocial behaviors, we detected smiles by the following signal processing algorithm. First, a 50–350 Hz band-pass filter was applied to extract the EMG signals by removing noise and outliers. Since each EMG signal is a superposition of multiple facial muscle activities, Independent Component Analysis (ICA) was applied to convert the filtered data into four independent signals to increase the saliency of each signal. Then, root-mean-squared averaging was applied to each independent component with a 100 ms averaging window. Finally, an ANN was trained using the analysis of human coders as a teaching signal to recognize the unobserved smiles of each participant. Among data of smile and no-smile, datasets having less noise and artifacts were used for training to evaluate the predictive performance on the testing set. This signal processing was performed by MATLAB R2017b (Mathworks, USA).

In previous studies, ANN has been used and suggested as a classifier to improve the classification accuracy for EMG signals (Maria et al., 2019; Singh et al., 2020). The performance was different depending on experimental settings. Other classification methods, such as Support Vector Machine and Convolutional Neural Network, were also suggested to increase classification accuracy (Toledo-Pérez et al., 2019; Bakırcǧlu and Özkurt, 2020). However, the Smile Reader showed high accuracy with ANN (Gruebler and Suzuki, 2014; Hirokawa et al., 2018). When an ANN was applied to detect positive facial expressions with the Smile Reader, the average Kappa Coefficient between human coders and the classifier was 0.95 (Gruebler and Suzuki, 2014), which shows highly identical inter-rater agreement. Therefore, we applied the ANN classification to detect the unobserved smiles of each participant.

## 3. Results

The results are organized in three subsections presented below. The first part, *Observation of Different Behaviors*, presents different aspects of participants in specific timings.

The second part, *Observation of Common Behavior Changes*, presents the common behavior changes witnessed before the children engaged in prosocial behaviors. We explored how their behavior changed following the robot's movements.

The third part, the *Behavior Model Framework*, presents the proposed Bayesian model framework for probabilistic inference with the observed variables. We implemented a model based on the data derived from the robot-assisted therapy. This model, however, is not conclusive due to the small sample size of this study, but it is representative of the method we propose that can be applied to similar robot-assisted therapies.

### 3.1. Observation of Different Behaviors

We observed smiles at specific timings to explore which timings could be more related to prosocial behaviors. Also, we explored whether different behaviors are observed between children with ASD and TD children, and the two sessions of children with ASD.

#### 3.1.1. Smiles and Prosocial Behaviors in the Walking Situation

**Figure 6:**
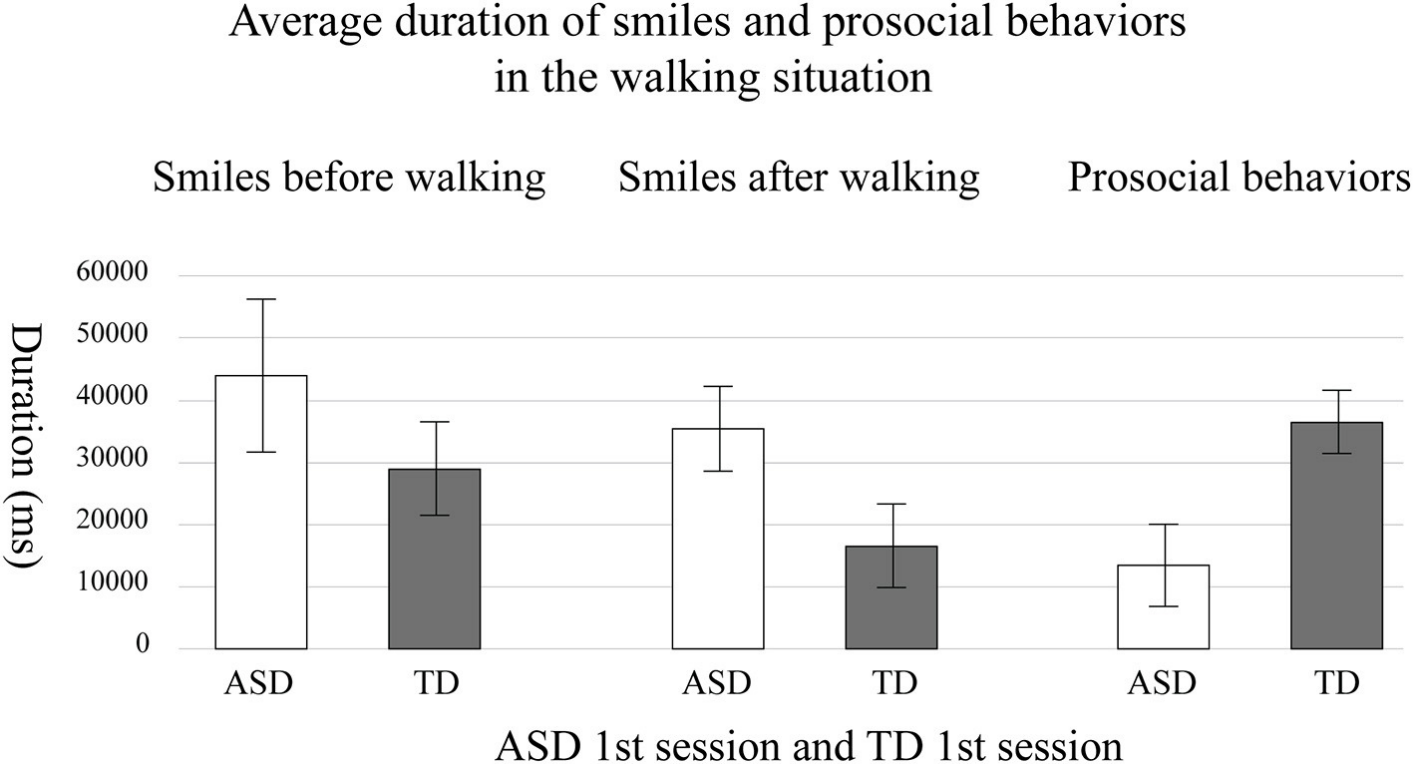
The average duration of the smiles and prosocial behaviors in the first session of children with autism spectrum disorder (ASD) and typically developing (TD) children. The error bar means standard error.

On average, children with ASD smiled longer than TD children in the walking situation (Tables 2, 3). TD children smiled the most when they entered the intervention room, then smiled less. On the other hand, TD children engaged in prosocial behaviors longer than children with ASD (Figure 6).

**Table 2:** The averaged smiles in the first and second session of children with autism spectrum disorder (ASD) (unit is seconds).

**ASD session 1**	**ASD session 2**	**Timing**
**Smile duration**	**Smile duration**	
**Mean ± SD**	**Mean ± SD**	
23.7 ± 17.9	24.9 ± 20.1	1 min after entering the intervention room
20.2 ± 12.9	24.6 ± 11.1	1 min before walking with the robot
35.5 ± 16.8	19.6 ± 15.2	1 min after starting walking with the robot
28.1 ± 11.9	24 ± 10.1	1 min before falling of the robot
7.7 ± 4.3	9.7 ± 4.3	While the robot was falling
43 ± 10.1	17.8 ± 8.7	1 min after the fallen robot was adjusted

**Table 3:** The averaged smiles in the first session of children with autism spectrum disorder (ASD) and typically developing (TD) children (unit is seconds).

**ASD session 1**	**TD session 1**	**Timing**
**Smile duration**	**Smile duration**	
**Mean ± SD**	**Mean ± SD**	
23.7 ± 17.9	20.4 ± 15.6	1 min after entering the intervention room
20.2 ± 12.9	8.5 ± 4.9	1 min before walking with the robot
35.5 ± 16.8	16.6 ± 16.3	1 min after starting walking with the robot
28.1 ± 11.9	17.7 ± 15.3	1 min before falling of the robot
7.7 ± 4.3	2 ± 3.5	While the robot was falling
43 ± 10.1	10.4 ± 7.8	1 min after the fallen robot was adjusted

**Figure 7:**
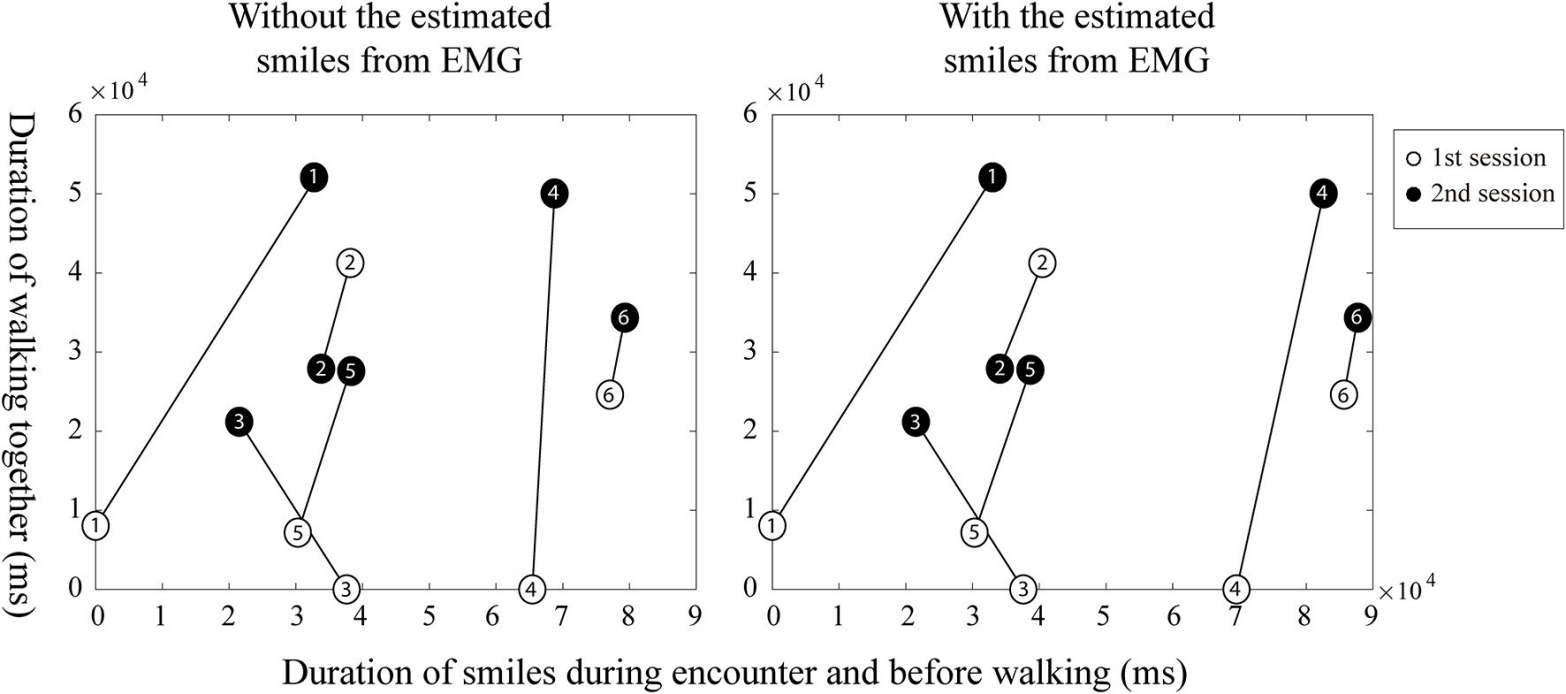
The possible relationships between smiles and prosocial behaviors from each child with autism spectrum disorder (ASD) in the walking situation.

When comparing the first and second session of children with ASD, each child with ASD showed different changes in the second session. Figure 7 indicates relationships between the duration of smiles and the duration of each participant's prosocial behaviors in the walking situation. The duration of smiles is the sum of smiles during the encounter and before walking together with the robot, as shown in segments in Figure 5a,b, which increased in the second session. The duration of prosocial behaviors is calculated as the sum of helping the robot walk. Empty symbols signify the first session; filled symbols signify the second session. The numbers in the symbols indicate the participant number of each child with ASD. Four children (ASD-P1, ASD-P4, ASD-P5, and ASD-P6) out of six children with ASD showed a longer duration of smiles and longer prosocial behaviors during the second session than during the first session. One child (ASD-P2) showed a shorter duration of smiles and a shorter prosocial behavior during the second session than in the first session. Another child (ASD-P3) showed an increased duration of prosocial behaviors but showed a decreased duration of smiles in the second session. Instead, the child started to sing a song before doing prosocial behaviors. The results imply the possibility of a positive relationship between smiles and prosocial behaviors in children with ASD.

#### 3.1.2. Smiles and Prosocial Behaviors in the Falling Situation

On average, children with ASD smiled longer than TD children in the falling situation (Tables 2, 3).

All children with ASD smiled at the robot during the falling moment in the first and second session. Among them, two children with ASD (ASD-P2 and ASD-P6) showed prosocial behaviors in the first session. Three children with ASD (ASD-P1, ASD-P2, and ASD-P5) showed prosocial behaviors in the second session.

In contrast, three TD children (TD-P1, TD-P2, and TD-P5) did not smile while the robot was falling. Among TD children, one child (TD-P4) immediately helped the robot stand up. Two TD children (TD-P2 and TD-P6) helped the robot after watching the fallen robot for ~10 s.

### 3.2. Observation of Common Behavior Changes

To investigate how behaviors change after the robot's movements and find a common series of behaviors before engaging in prosocial behaviors, we observed the behaviors of each participant 10 s before prosocial behaviors. If smiles are observable and other behaviors follow the smile, we might be able to predict behaviors after smiles. Also, if smiles are triggered by a robot, we might be able to arouse timely smiles and facilitate prosocial behaviors using a robot.

The observation was based on a total of 36 cases of robot walking and falling situations, including 12 cases of children with ASD in the first session, 12 cases of children with ASD in the second session, and 12 cases of TD children in the first session. We observed four types of common cases.

#### 3.2.1. Before Walking of Robot

Case A: Cases of children who showed smiles and prosocial behaviors.

ASD-P1, ASD-P2, ASD-P4, and ASD-P6 showed smiles toward the robot after watching the robot's movements, such as nodding and reaching out its arms. After smiling, they maintained their head direction toward the robot, went closer to the robot, then showed prosocial behaviors voluntarily. In the case of ASD-P6, the child showed the same pattern of behaviors both in the first and second session.

We found similar interactions from TD-P2, TD-P4, and TD-P5. Children, who smiled and maintained their head direction toward the robot, went closer to the robot, and showed prosocial behaviors voluntarily. The smiles were triggered by the robot's movement or observation of interactions between a parent and the robot.

On the other hand, ASD-P2 showed smiles toward the robot after watching the robot's nodding. However, the child's head direction changed to toward their own body, and the child started to move their own fingers without smiling. When the child was focusing on his fingers, his parents tapped his back two times and suggested walking with the robot. The child looked at his parents and then stood up to hold the robot's hands.

Case B: Cases of children who did not show smiles and prosocial behaviors.

ASD-P3 in the first session did not smile after watching the robot's nodding and standing up, and did not show prosocial behaviors. The robot's movements made the child move the head toward the robot temporarily; however, the child did not maintain the head direction. The child looked at the therapist's camera and made a V shape with fingers in the first session.

Case C: Cases of children who showed smiles but did not show prosocial behaviors.

ASD-P4 in the first session smiled toward the robot after watching the robot's standing up. However, the child did not maintain the head direction. The child started to smile toward the parents and went closer to them.

Case D: Cases of children who did not show smiles but showed prosocial behaviors.

Total eight cases from children with ASD and TD children did not smile after watching the robot's movements but showed prosocial behaviors. Before engaging in prosocial behaviors, they received a parent's help or additional direction from the therapist. When their head direction was toward the robot, the child started to follow that direction.

#### 3.2.2. During Falling of Robot

Case A: Cases of children who showed smiles and prosocial behaviors.

In the five cases, children with ASD smiled toward the robot when it was falling and then they moved closer to the robot. Their head direction was continuously directed toward the robot. The children smiled toward the robot before starting to engage in prosocial behaviors.

TD-P2, TD-P4, and TD-P6 also showed smiles and prosocial behaviors. However, they showed different aspects of behaviors that were not observed in children with ASD. TD-P4 and TD-P6 looked at the therapist after doing prosocial behaviors. TD-P2 did not show smiles when the robot was falling. However, the child looked at the therapist after the robot fell and asked the therapist if helping the robot is allowed. Then the child smiled toward the robot before engaging in prosocial behaviors.

Case B: Cases of children who did not show smiles and prosocial behaviors.

In the six cases, children with ASD released the robot's hands and became distant from the robot when the robot was falling. The head direction was continuously directed toward the robot.

On the other hand, TD-P1 and TD-P3 looked at the therapist after distancing from the robot. TD-P5 watched the robot's falling while sitting behind and holding onto a parent. The head direction of this child was continuously toward the robot, but this child did not show any different facial expressions or body movements after seeing the robot falling.

Case C: Cases of children who showed smiles but did not show prosocial behaviors.

ASD-P4 smiled toward the robot when the robot was falling but did not show prosocial behaviors both in the first and second session. In the first session, the child started to smile while looking around the intervention room and did not move closer to the robot. In the second session, the child smiled toward the robot when the robot was falling, then continuously smiled toward the robot. However, the child did not move closer to the robot.

Case D: Cases of children who did not show smiles but showed prosocial behaviors.

None of the participants' behaviors fell into this category.

### 3.3. Behavior Model Framework

We propose a probabilistic model framework based on the observation of the behavior changes. We particularly applied a Bayesian approach to be able to include the uncertainty of variables and flexibly represent changes in the relationships among variables (Kumano et al., 2015; Mózo, 2017). Also, using Bayesian methods is recommended by American Statistical Association because it can provide the magnitude of treatment in a clinical setting with probabilistic inference (Ronald et al., 2019). Therefore, we first observed a common series of behaviors from all participants, and then we represented the behavior changes with a Bayesian approach. If we can find consistent patterns, it could be used as a framework for future robot-assisted therapy.

#### 3.3.1. A Series of Behaviors

**Figure 8:**
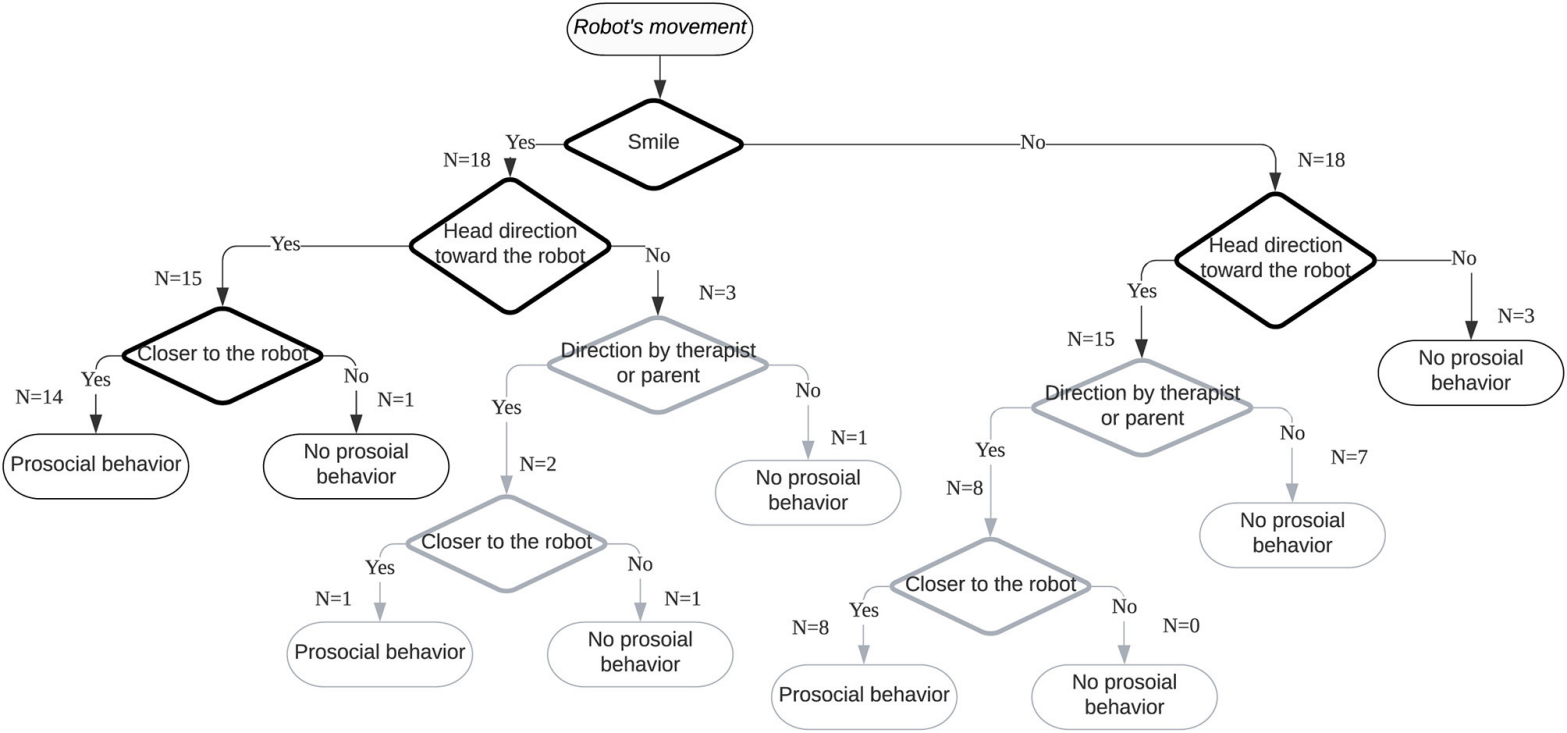
A flowchart of child–robot interactions before prosocial behaviors.

In this study, a series of behaviors were observed from both children with ASD and TD children before engaging in prosocial behaviors. These common behavior changes are expressed in a flowchart (Figure 8).

We identified three types of smile triggers during the robot-assisted activities. Most children smiled after the robot exhibited movements, such as nodding and reaching out its arms. Walking and falling of the robot were also triggers for smiles. The second trigger type was related to the child expecting robot movements. In this research, two children smiled when they started interacting with the robot. The third trigger was observing the robot's movements. One child smiled after observing an interaction between the robot and a parent. All three smile triggers were related to the experience of watching the robot's movements.

After the robot's movement, we observed that smiling, heading toward the robot, and approaching the robot might be connected factors in the time series with prosocial behaviors. Before 10 s of doing prosocial behaviors, the three types of behaviors kept changing. However, once a smile was detected, when the head direction was toward the robot, approaching the robot and doing prosocial behaviors occurred. In particular, smiles toward the robot preceded voluntary prosocial behaviors. This finding indicates that if a child with ASD shows a smile, heads toward a robot, and approaches the robot, there is a high probability that prosocial behaviors will be performed.

**Figure 9:**
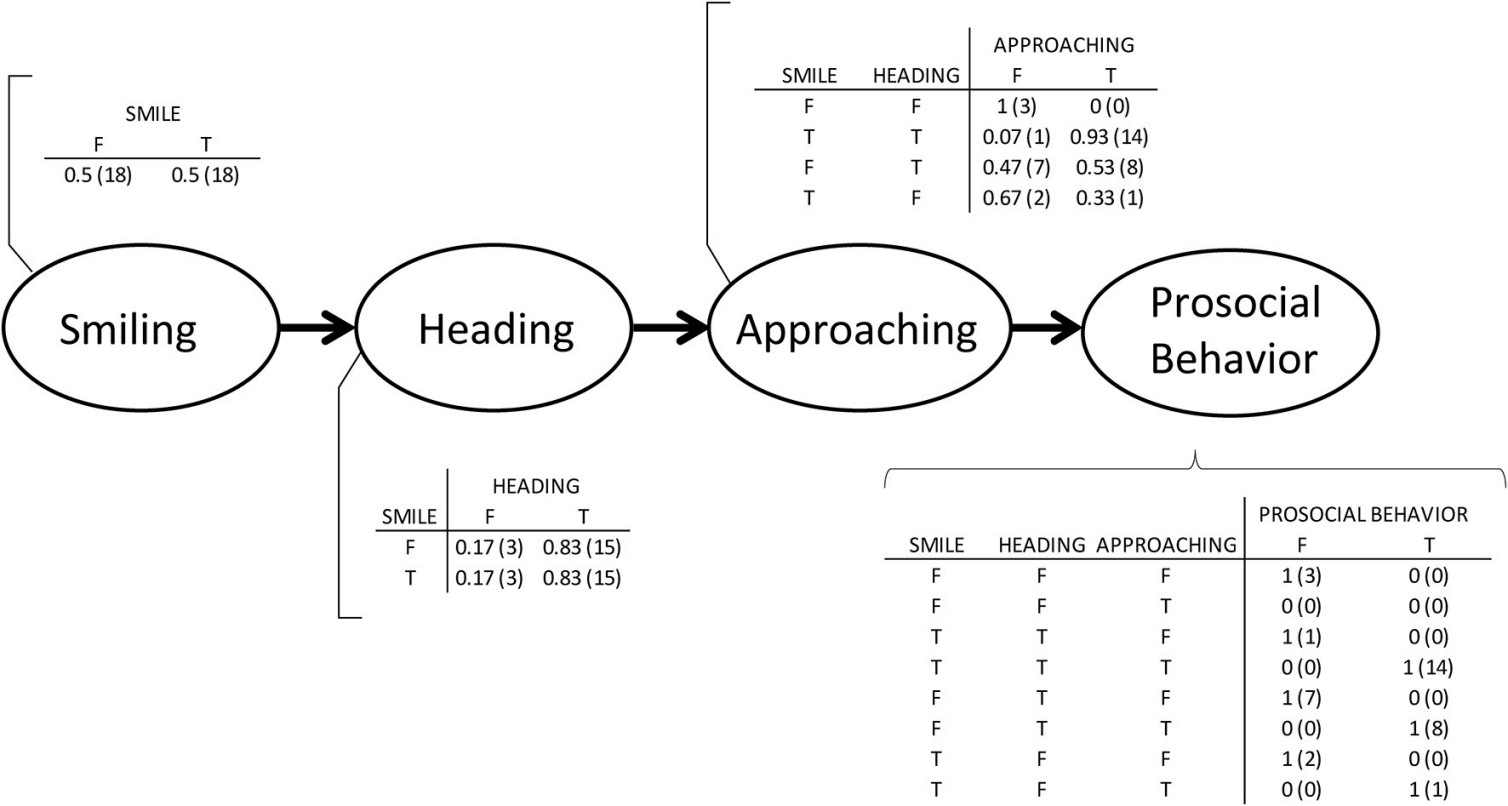
A proposed Bayesian network with conditional probability tables. The number of cases is in parentheses.

This Bayesian framework with conditional probability tables represents the relationships among the four variables (Figure 9). The probability of each node was acquired from the 36 cases of video observation, which are 10 s before prosocial behaviors in each. Therefore, the probability of smiles when children showed prosocial behaviors may be useful to predict the likelihood of prosocial behaviors when smiles are observed. This conditional probability can be expressed by Bayes' theorem as follows:

(1) $$\begin{array}{r} P\left(PB|S\right)=P\left(S,PB\right)/P\left(S\right) \end{array}$$

*PB* denotes doing prosocial behaviors, and *S* denotes smiling. When the two variables are assumed to be independent, the likelihood of prosocial behavior given a smile can be calculated. From the 36 cases of video analysis, the probability of a smile was 0.5; the probability of prosocial behavior was 0.64. When participants engaged in prosocial behavior, the probability of smiles before their prosocial behavior was 0.42. Table 4 shows the joint probability of smiles and prosocial behaviors, and includes both voluntary prosocial behaviors and those directed by a therapist or a parent. Therefore, we may predict the likelihood of prosocial behavior given a smile:

(2) $$\begin{array}{r} P\left(PB|S\right)=0.42/0.5=0.84 \end{array}$$

The likelihood of prosocial behavior given a smile was 84%, only if the probability of prosocial behavior is known, and then the probability of smile before prosocial behavior is known.

(3) $$\begin{array}{r} P\left(PB|\bar{S}\right)=0.22/0.5=0.44 \end{array}$$

On the other hand, the likelihood of prosocial behavior given no smile was 44% only if the probability of prosocial behavior is known and the probability of no smile before prosocial behavior is known. Here, $\bar{S}$ denotes no smiling. In this study, the probability of no prosocial behavior was 0.08 after smiling; the probability of prosocial behaviors after smiling accounted for 66% of the total prosocial behaviors. This result signifies that we could predict prosocial behaviors by analyzing smiles and that we could facilitate prosocial behaviors by arousing smiles. If a child does not smile, interactions with a robot will be helpful. Such intervention may result in further interactions between the child and the robot that trigger smiles.

**Table 4:** Joint probability of smiles and prosocial behaviors from 36 cases of participants.

	**Prosocial behavior**		
**Smile**	**Yes**	**No**	**Total**
Yes	0.42	0.08	0.5
No	0.22	0.28	0.5
Total	0.64	0.36	1.0

#### 3.3.2. Model Validation in the Robot-Assisted Therapy

To evaluate the estimation with the Bayesian model, we used leave-one-out cross-validation. With this method, we can validate the model using the small sample, as the collected data can be used for both training and testing (Russell and Norvig, 2010). Also, this method can be used to validate the predictive accuracy of the Bayesian model (Vehtari et al., 2017). The entire dataset was used for training in this model, except for data from one participant that was used for testing. This process was repeated for all participants one by one with all combinations of the predictors. Then, the accuracy of each predictor was averaged. The selected predictors were prosocial behavior, smiling, heading toward the robot, and prompting by a therapist or a parent. Approaching toward the robot was not selected as a predictor because prosocial behaviors always happened when smiling, heading, and approaching occurred with the sample data. Also, we included prompting in this model considering that the therapeutic setting in this study is to assist the therapist or the parent.

**Figure 10:**
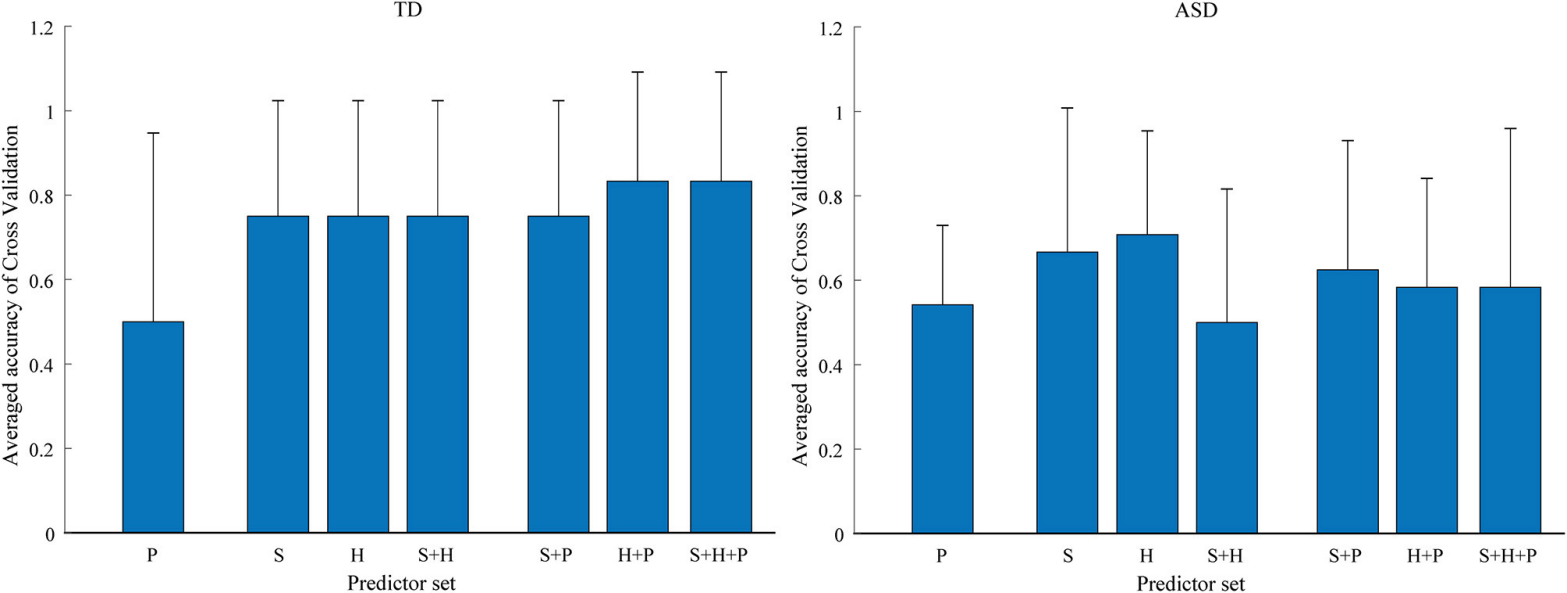
The accuracy of each predictor and the combinations of predictors for typically developing (TD) children and children with autism spectrum disorder (ASD).

Figure 10 shows the accuracy of each predictor and the combinations of predictors. *S* denotes smiling. *H* denotes heading toward the robot. *P* denotes prompting by the parent or the parent. + means combinations of two or three predictors.

The results show that the prosocial behaviors of children with ASD and TD children were predicted differently. For TD children, the highest accuracy of prediction was found when using the combination of smiling, heading, and prompting as a predictor. This finding indicates that prosocial behaviors could be predicted with over 80% accuracy on average by detecting smiling, heading toward the robot, and then prompting. The prediction accuracy was the lowest when only prompting was used. However, prosocial behaviors were facilitated when prompting was provided after smiling or heading toward the robot. Also, 78% of prediction accuracy was achieved with only smiles or only heading toward the robot as predictors, suggesting that we could predict prosocial behavior of TD children with the single factor of smiling or heading toward someone.

On the other hand, for children with ASD the highest accuracy of prediction was found when heading toward the robot was used as a predictor. Prosocial behaviors could be predicted with 70% accuracy, on average, using this single predictor. Smiling was the second most predictive variable, with a prediction accuracy of 65%. The prediction accuracy of prompting was low both when it was considered as a single factor and when it was combined with other factors. These results indicate that children with ASD showed more voluntary prosocial behaviors without prompting compared to TD children. Also, we could predict the prosocial behavior of children with ASD with the single factor of smiling or heading.

Although the prediction accuracy of heading is higher than smiling for children with ASD, detecting smiling can provide useful information for personalized robot-assisted therapy. In this study, all the children with ASD who smiled after watching the robot's movement showed prosocial behaviors voluntarily without prompting by the therapist or the parent. In contrast, all the children with ASD who did not smile after the robot's movement yet showed prosocial behaviors received prompting by a therapist or a parent. This finding signifies that smiling might be a signal of voluntary prosocial behaviors. With this model, if smiling does not appear, we could predict prosocial behaviors by detecting heading toward the robot. Therefore, it is possible for a therapist to control the robot to arouse smiles to facilitate voluntary prosocial behaviors. Also, a therapist can decide the timing of prompting to make children with ASD practice prosocial behaviors.

## 4. Discussion

We explored the potential of personalized robot-assisted therapy based on smile analysis. Particularly, we explored whether smiles can be a potential key factor in predicting prosocial behaviors toward the robot in the therapeutic setting. Each child experienced the walking and falling of a NAO robot. The main findings are as follows.

First, we observed the changes in the smiles and prosocial behaviors of each child with ASD. When the duration of smiles increased when entering the intervention room and before walking, five out of six children with ASD engaged in more prosocial behaviors. Likewise, in the falling situation, three children with ASD showed prosocial behaviors in the second session. They smiled more than in the first session when the robot was falling. Other children, who showed a shorter duration of smiles in the second session, did not help the robot. It suggests that positive affect can be related to prosocial behaviors. Also, it might be helpful to arouse positive affect before intervention for the target behavior.

Second, there were behavioral differences between children with ASD and TD children in the two social situations. Overall, children with ASD smiled more and exhibited fewer prosocial behaviors than TD children. Children with ASD easily responded to the robot's movements by smiling or moving their bodies. It suggests that an interaction with a robot can induce immediate behaviors in children with ASD. On the other hand, TD children smiled the most during the first moment with the robot and then smiled less. This result might indicate that TD children lost interest in the robot after the first encounter. Otherwise, it is possible that they showed fewer smiles but maintained a positive affect for longer than children with ASD.

There was also a difference in head direction behavior between children with ASD and TD children in the falling situation. While all children with ASD continuously headed toward the robot after the robot fell, all TD children headed toward their caregiver or a therapist. It should be noted that the falling of the robot occurred unexpectedly and did not include a cue providing additional directions. Hence, the observation that these children responded to the falling by heading toward an adult can be explained by typical social referencing (DeQuinzio et al., 2016). TD children tend to refer to the verbal and nonverbal behaviors of a parent or a caregiver in unfamiliar social situations. In this research, TD children required directions or confirmations from adults in the falling situation.

Aside from the difference in the duration of smiles and prosocial behaviors, we observed that the two groups of children exhibited common behaviors before engaging in prosocial behaviors. An analysis of the video fragments taken 10 s before prosocial behaviors revealed four types of behaviors that might be connected in a time series: Smiles were followed by heading toward the robot, approaching the robot, and voluntary prosocial behaviors. Based on these findings, we suggested a Bayesian model for predicting prosocial behaviors and validated the model using leave-one-out cross-validation. Using this model, smiling could predict prosocial behavior of both children with ASD and TD children with an accuracy of at least 65%. When smiling is not observed, heading toward the robot predicted prosocial behaviors prompted by a therapist or a parent. Children with ASD showed more voluntary behavior changes by the robot compared to TD children. All children with ASD, who showed smiling after watching the robot's movements, engaged in prosocial behaviors without prompting, suggesting that simply arousing smiles by having the child watch the robot might facilitate prosocial behaviors individually.

This research was an exploratory study in a therapeutic setting examining the use of a robot to assist a therapist. Therefore, there are several limitations regarding the applicability of our results in other settings.

First, the number of sessions and cases was limited. Although children with ASD participated in a total of four sessions, and TD children participated in a total of three sessions, the maximum sessions for this research included two sessions with children with ASD and one session with TD children. Due to this limitation, statistical tests between the two groups could not be performed. In addition, some of the children with ASD experienced more therapy sessions between the two selected sessions, and this might have affected the results. Therefore, data availability for research should be considered when selecting the types of prosocial behaviors in the next research.

Second, the effects of playing with the robot and the effects of prosocial behaviors on smiles were not investigated. Although the duration of smiles before prosocial behaviors included playtime, future research should investigate how play affects mood or emotions toward the robot. Also, it is possible that prosocial behaviors toward the robot affected the next smiles, and the smiles affected prosocial behaviors. This cyclic chain of behaviors should be explored in future research.

Third, the analysis of different types of facial expressions was limited. In this research, positive affect and smile were focused. Therefore, we observed the changes of facial muscles related to positive affect. However, detailed smile analysis might capture different behavior patterns. In future research, better methods for capturing facial expressions should be considered.

Fourth, there were motion artifacts caused by the movements of each participant during the robot-assisted activities. Although the high accuracy of classifying smiles was reported in previous studies and the method was followed in this research, the recorded EMG of each participant included a different amount of motion artifacts. Therefore, we detected more smiles from EMG signal processing, but there is a possibility of including both actual smiles and artifacts. It should be considered to reduce artifacts when recording and analyzing the EMG of entire sessions.

Another limitation is the lack of detailed profile data of children with ASD. As they were recruited and identified with ASD through the Institute for Developmental Research, the standardized tests for their diagnosis and the diagnostic results could not be reported in this paper. Also, the age variance of participants was high. It was not confirmed whether their developmental status is comparable. The high age variance could affect the behaviors toward the robot. These limitations should be considered when designing the experiments for future research to differentiate applicable levels of child development.

Despite the limitations of this research, the results show that more prosocial behaviors toward the robot were observed when the smiles of a child were observed. This result highlights the potential benefits of smile analysis and the use of a robot to facilitate prosocial behaviors in children with ASD. Considering that smiles might be a signal of prosocial behaviors, personalized therapy for children with ASD could include analyzing smiles, predicting prosocial behaviors, and inducing smiles. Therefore, if it is possible to predict prosocial behaviors consistently based on the proposed Bayesian model, this theoretical framework will enable future robot-assisted interventions to tailor a robot's behaviors according to smiles and other related behaviors of each child with ASD. Moving forward from the previous studies that investigated the effects of robot-assisted therapy (Zheng et al., 2016; Cao et al., 2019), this exploratory research suggested a framework of how prosocial behaviors could be predicted by smiles and how behavior changes could be aroused by a robot. Furthermore, it is expected to apply this approach to other smile-related behaviors, such as emotional empathetic behaviors (Sonnby-Borgström, 2002; Deschamps et al., 2014; Telle and Pfister, 2016).

## 5. Conclusions

In this exploratory research, we studied how prosocial behaviors of children with ASD could be facilitated in robot-assisted therapy based on smile analysis. In this research, we observed that specific timings of smiles and prosocial behaviors were increased on average in the second session of children with ASD. Second, we observed that TD children smiled shorter, but they engaged in prosocial behaviors longer than children with ASD. Third, the robot's movements could trigger the smiles of both children with ASD and TD children. Fourth, voluntary prosocial behaviors occurred after smiling. Fifth, when a smile was not observed, prosocial behaviors of children with ASD were prompted by a therapist or a parent. Lastly, we could predict prosocial behavior of both children with ASD and TD children with the single factor of smiling or heading by applying the proposed Bayesian model. These observations indicate that prosocial behaviors might be facilitated by inducing timely smiles. One way can be arousing smiles before starting the therapy stage for practicing prosocial behaviors. Another way is to predict the next prosocial behaviors with the proposed Bayesian framework and control a robot to arouse smiles timely. In this research, once a smile appeared, both children with ASD and TD children engaged in prosocial behaviors. When considering that children with ASD responded to a robot's movements with more smiles than TD children, this framework could be applied to personalized robot-assisted therapy for children with ASD.

In future research, the Bayesian model will be applied to another therapy with different participants and different social situations that arouse prosocial behaviors. If the same patterns are observed in such future research, the model can become a framework for robot-assisted therapy facilitating prosocial behaviors. Additionally, the possible array of robot movements that could trigger smiles will be investigated in more detail in the next phase of our research. Furthermore, we will investigate whether this smile analysis can be expanded to other smile-related behaviors. We expect to develop an automated system with this Bayesian framework that can detect the smiles of a child with ASD, anticipate the child's prosocial behaviors, and provide therapeutic interactions with the child in real time, thus providing therapists with more resources to focus on sophisticated behavior changes.

## Data Availability Statement

The datasets, video annotation in robot-assisted therapy, presented in this study can be found in an online repository at: doi: 10.7910/DVN/2VLYY8.

## Ethics Statement

The studies involving human participants were reviewed and approved by Ethical Committee established by the Aichi Human Service Center. Written informed consent to participate in this study was provided by the participants' legal guardian/next of kin. Written informed consent was obtained from the minor(s)' legal guardian/next of kin for the publication of any potentially identifiable images or data included in this article.

## Author Contributions

SK, MH, SM, and KS: conceptualization and methodology. SK and AF: data curation. SK: formal analysis, visualization, and writing. SK and KS: funding acquisition and project administration. SK, MH, and AF: investigation. KS and AF: resources. MH: software. KS, MH, and SM: supervision. All authors contributed to the article and approved the submitted version.

## Conflict of Interest

The authors declare that the research was conducted in the absence of any commercial or financial relationships that could be construed as a potential conflict of interest.
